# Ushering in the New Toxicology: Toxicogenomics and the Public Interest

**DOI:** 10.1289/ehp.7732

**Published:** 2005-03-24

**Authors:** John M. Balbus

**Affiliations:** Environmental Defense, Washington, DC, USA

**Keywords:** bioinformatics, computational toxicology, metabolomics, metabonomics, microarrays, predictive toxicology, proteomics, toxicogenomics, transcriptomics

## Abstract

New scientific tools spawned by the genomics revolution promise to improve our ability to identify causative factors in human diseases. But as these new tools elucidate the complex interactions between chemical toxins and biologic systems, the strain on traditional ways of understanding toxic effects grows. Despite major advances in the science and technology of these new toxicogenomics tools, scientific and political complexities threaten to delay the use of toxicogenomics to further the public interest or—worse—to advance its use initially to weaken the regulation and safety of widely used chemicals. To gain further insight into the scientific and political landscape of the new toxicology, we interviewed 27 experts from a variety of disciplines and sectors. Interviewees expressed widespread agreement that the new toxicology promises a significant increase in the amount of information available on toxic effects of chemicals. But the interviews show that the promise of the new toxicology will be realized only if technical and political obstacles can be overcome. Although scientific rigor is necessary for the new toxicology to move forward, the scientific and public-interest communities must ensure that inappropriate definitions of rigor, as well as proprietary interests, do not create unnecessary barriers to more effective public health protection.

New scientific tools spawned by the genomics revolution promise to improve our ability to identify causative factors in human diseases. These tools are expected to allow more rapid screening of chemicals for toxic effects and to provide mechanistic insight into a greater range and earlier stage of adverse outcomes associated with chemical exposures. Greater reliance on computer-based models has already brought remarkable advances in our ability to predict disease progression. For example, Petricoin and Liotta (2003) and Petricoin et al. (2002) have demonstrated a diagnostic screening test based on serum protein patterns for early detection of ovarian cancer. But the reliance on computers may make it more difficult for scientists trained in traditional toxicology to integrate this new knowledge into existing paradigms. In addition to barriers within the scientific community, the emergence of these new technologies is taking place in a political context that involves a variety of stakeholders with separate agendas. Thus, despite major advances in the science and technology of these new toxicogenomics tools, these scientific and political complexities threaten to delay the use of toxicogenomics to further the public interest or—worse—to advance its use initially to weaken the regulation and safety of widely used chemicals. In this article we highlight three important issues in the development of toxicogenomics and then report on a series of expert interviews that give additional insights into these and other critical questions.

## Replace, Augment, or Refine?

How will toxicogenomics be developed and incorporated into testing and regulatory regimes? The often-stated promise of toxicogenomics techniques is that they will improve the screening of chemicals for toxicity by being faster, cheaper, more accurate, and more comprehensive than existing methods. But this promise is likely to be realized only after a period of relatively expensive and deliberate test validation and generation of the massive reference databases needed to make rigorous conclusions about test results. Without proper study design, appropriate use of controls, and multidisciplinary development of standardized methods, acceptance of new screening tests will be slow. In the interim, the power of toxicogenomics is likely to be applied piecemeal, with specific and often proprietary toxicogenomics assays developed and applied primarily to address the problems faced by regulated industries.

The pharmaceutical industry has been capitalizing on the strengths of toxicogenomics to screen compounds for potential toxicity. Large pharmaceutical companies have invested in enormous databases of genomic responses to known toxins and complex pattern recognition software programs to screen test data against these reference compounds (Hood 2003). This reflects the enormous savings to the pharmaceutical industry from early identification of the potential toxicity of a new product as well as the fact that the drug development process has a significant backstop to toxicogenomics screening in the form of extensive required clinical testing. This backstop allows screening for a limited set of toxic end points; early identification of drugs causing the limited set of toxicities is still cost-effective, whereas the subsequent rigorous clinical testing helps protect the public by detecting other types of toxicity.

The chemical industry, on the other hand, does not face any specific regulatory testing requirements for new products. The Toxic Substances Control Act of 1976 (TSCA) (1976) stipulates that all known toxicity information be disclosed in a premanufacture notice, but it does not require that any specific testing be performed before manufacture and marketing of a new chemical product. Thus, the chemical industry has less of a regulatory incentive to prescreen chemicals under development for potential toxicity. Although the stated public positions of the American Chemistry Council (ACC) and the Chemical Industry Institute of Toxicology Centers for Health Research (CIIT) describe a desire to broaden the understanding of potential health risks of chemical products, the regulatory context and incentive structures are leading to a focus on revising risk assessments of existing products that the industry believes overestimate true risks (e.g., Greenlee et al. 2003). Thus, the CIIT is applying toxicogenomics and systems biology approaches to compounds such as formaldehyde and chloroform in order to more fully elucidate cancer mechanisms and potentially demonstrate nonlinearity of cancer dose responses. The CIIT asserts that systems biology will provide a “valuable payback” by reducing uncertainty and that reduction in uncertainty will affect environmental standard compliance costs, presumably by allowing more relaxed exposure standards. The ACC’s Long-Range Research Initiative, in response to recent funding cuts, has redirected its academic program away from exploratory toxicogenomics and systems biology work but is continuing to fund the CIIT to perform more focused work on existing chemicals (MacKenzie 2004). For now, the stronger incentive for the chemical industry to use toxicogenomics to improve mechanistic understanding and reduce uncertainty factors rather than to develop new, more powerful screening tools suggests that the public need for better screening tools will have to be met elsewhere.

## Phenotypic Anchoring: Scientific Rigor or Scientific Shackling?

With the massive number of data points now being monitored in gene and protein expression assays, some stakeholders are concerned that changes in gene expression or protein levels will be mistakenly interpreted as related to adverse effects when in fact they are benign. Within the chemical industry, some are calling for linking toxicogenomics results to traditional toxicology tests (e.g., Henry 2002). The implication is that changes in gene expression should not be considered adverse unless they can be directly related to outcomes observed in traditional toxicology tests.

Although, on the face of it, this may seem like a reasonable way to inject rigor into a complicated process, placing overly strict limitations on interpretation of gene expression data ensures that only the end points now assessed by traditional toxicology tests will be the end points considered in the future. One of the greatest promises of toxicogenomics is the ability to assess toxicity more comprehensively, picking up more subtle changes than may be detected by histopathology or other traditional detection methods. Tying the interpretation of toxicogenomics testing so strictly to traditional toxicity tests would keep this promise from being realized because information gained could be no more comprehensive than current test methods. Such scientific shackling of toxicogenomics approaches must be prevented if the new technologies are going to provide their maximal benefits.

## Toxicogenomics Meets Toxicogenetics

Can the study of the toxic effects of chemicals be separated from the study of the genetic mechanisms underlying variations in individual susceptibilities to those chemicals? In the pharmaceutical arena, the observation that a new drug for small cell lung cancer works miraculously, but only for around 10% of those affected, demonstrates the power to identify critical variations in genetics and raises the ethical issue of dividing populations into those who benefit from new technology and those who do not (Lynch et al. 2004). Molecular epidemiology studies similarly show that people with particular genotypes are at increased risk of adverse health outcomes from toxic exposures. Various metabolic polymorphisms have been shown to convey small differences in risks for environmental exposures (Kelada et al. 2003); more extreme examples, whereby rare genotypes or combinations of genotypes convey greatly increased risks, are certainly possible. Current regulatory regimes inconsistently address interindividual differences in susceptibility. The Clean Air Act of 1990 (1990) (and the Safe Drinking Water Act Amendments of 1996 (1996) require explicit consideration of sensitive subpopulations in setting standards. TSCA, on the other hand, is silent on the subject of individual susceptibility. Inadequate ethical and societal frameworks for addressing susceptibility may lead to public backlash against generating toxicogenetic information or, worse, inappropriate or discriminatory use of such information.

To gain further insight into the scientific and political landscape of the new toxicology, we interviewed 27 experts from a variety of disciplines and sectors. Their views on the current status of the different fields within toxicogenomics, where they are going, and what the barriers are to fulfilling their potential provide an opportunity to compare and contrast different stakeholder viewpoints and suggest roles for the public-interest science community in promoting beneficial applications of toxicogenomics.

## Materials and Methods

Interviewees were selected to represent five different sectors involved in toxicogenomics: government researchers, government regulators, academic researchers, private-sector scientists, and public-interest scientists. Of 34 experts initially identified for interviews, two declined, saying they were not deeply enough involved in the field to speak about it, and five could not be reached. The author and one assistant conducted the interviews by telephone, using a structured set of questions as a guideline. We recorded and took notes during the interviews and have complete transcripts of each interview, except for four that were not fully transcribed because of problems with recording.

We analyzed the transcripts by thoroughly reviewing the text and identifying important statements. Although the categories of responses were partly determined by the specific questions, the answers often overlapped with other issues. The categories of response we used were *a*) general challenges to chemical regulation and safety, *b*) impacts of toxicogenomics on chemical regulation and safety, *c*) applications closest to implementation, *d*) applications furthest from implementation, *e*) barriers to implementation, *f* ) use of animals in testing, and *g*) actions that should be taken by the environmental public-interest community.

Comments were assigned to one of these categories and put into a separate database to facilitate comparison of the responses. The responses then were grouped according to the respondent’s sector.

## Results

### Question 1—What is the greatest challenge facing chemical regulation and safety?

Most respondents mentioned long-standing limitations of traditional toxicologic testing, specifically the difficulties associated with using high doses in animals to study low-dose effects in humans. In addition, many of the respondents mentioned the inability of traditional toxicologic testing to address issues of mixtures and to differentiate easily between genotoxic and nongenotoxic carcinogens. Finally, many respondents referred to the fact that the large number of chemicals used in commerce precludes obtaining enough data to assess their risks, at least using current testing methods.

Several respondents pointed out other challenges of the current system. One respondent described the greatest challenge as persuading public-health professionals to recognize (at least in that person’s opinion) that the toxicity of common pharmaceuticals greatly exceeded the toxicity of environmental chemicals and that more efforts should be made to reduce the public-health burden of adverse drug reactions. Another felt that the need to overcome the differing paradigms for cancer versus noncancer end points was a significant problem. Another respondent pointed to the lack of good exposure data and generally poor risk communication as problems.

Many respondents spoke about the same uncertainties from different perspectives. One academic talked about reaching the right balance in chemical regulation (presumably balancing need to protect public health with needs to allow commerce to continue), whereas some in academic research and the private sector were more concerned that the current use of uncertainty factors was unscientific and overly protective.

### Question 2—Will the impacts of this new technology on chemical regulation and safety be positive or negative?

Most of the respondents felt that the development of new technology would be positive because more information about biologic effects of chemicals would allow more effective controls. Many said the most positive impact would be that toxicogenomics could help avoid the need for dose and species extrapolation and reduce the use of uncertainty factors. One government researcher referred specifically to obtaining information about controversial beneficial effects of low-dose exposures to compounds that are toxic at a higher dose. Several also mentioned that toxicogenomics methods might provide insights into whether mixtures of chemicals produce additive, synergistic, or antagonistic effects.

Several perspectives regarding potential scientific benefits emerged. One respondent pointed out that compared with traditional toxicology tests, which are generally limited in the number of end points that can be assessed, toxicogenomics assays have a greater ability to measure multiple dose–response curves for multiple end points and multiple points in time. This reinforces that much more information will be available from a given experiment, with both positive consequences (better understanding of actual biologic responses) and negative ones (difficulties of handling and interpreting massively increased amounts of data). Two respondents from industry pointed to the benefit of greater confidence in the safety (and lowered liability risk) of products. Concerning potential negative impacts, several respondents, including those within the industry sector, expressed concern that the technologies were being forced too soon. Negative consequences included misinterpreting the data and making regulatory decisions before there was sufficient scientific certainty.

### Question 3—What applications are closest to implementation?

Several applications and types of information were cited as feasible in the near term. Indeed, respondents noted several applications that were presently under way and had been used to generate data already published. Many felt that gene and protein arrays would yield useful information about mechanisms and/or modes of action in the near future. A few qualified this by limiting the near-term applications to those related to mechanisms of action that are already fairly well characterized, especially those that are mediated with known receptors.

Many respondents, especially those in the pharmaceutical industry, mentioned the ability to screen compounds for some types of toxicity early in the drug development process. An interesting area of divergence was whether these same techniques could be applied to chemicals in general. Several respondents from the academic and government research sectors felt that the ability to obtain hazard-identification information from gene expression assays, at least for some types of hazards, was close at hand. In the private sector, some felt that such ability was also close at hand, but others felt that lack of reproducibility of assays prevented any effective use of toxicogenomics data. For some respondents, differences of opinion about the feasibility of toxicogenomics for chemical screening were related to different concepts of what that entailed. Those that had in mind screening for recognized types of toxicity on chemicals with some well-characterized structural analogs generally felt that such screening was close at hand. Respondents who felt that screening was further in the future generally conceptualized such screening as involving completely unknown chemicals and screening them comprehensively for any type of toxicity.

Many respondents felt that the ability to understand the importance of polymorphisms for susceptibility to toxic compounds was near. Different respondents, however, often had different applications in mind when speaking of susceptibility. Some were referring to the ability to understand and predict an individual’s adverse reactions to pharmaceuticals, whereas others were referring to identifying susceptibility factors for toxic exposures in populations and individuals. There was a further distinction between the ability to identify specific genetic susceptibility factors for adverse outcomes from specific drugs or toxic exposures and the ability to use a more comprehensive genetic profile to either tailor pharmaceutical interventions or characterize a person’s overall susceptibility to a variety of toxic exposures. Although many respondents believed genetic susceptibility to be a near-term prospect, those who were referring to the more comprehensive application of genetic susceptibility factors felt that this application would not be available for many years. Sector affiliation did not correlate with the views on this topic, with private and public sector respondents represented on both sides of the disagreement.

### Question 4—What applications are furthest from implementation?

Many respondents considered the use of toxicogenomics data quantitatively within risk assessments to be far from implementation. Reasons for this included the technical difficulty of getting the assays to provide reliable, reproducible, quantitative dose–response information, as well as the political and social difficulties of convincing regulatory agencies to change their practices. The ability to analyze complex mixtures and long-term low-dose effects was also considered unlikely to be realized soon.

A number of respondents placed the ability to model complex biologic systems as being at least 5–10 years away. This theme encompassed the potential to link gene expression changes to specific biologic pathways that includes higher levels of biologic organization, from protein expression through pathology, as well as the promise of *in silico* modeling and the ability to test chemicals for toxicity without the use of animals. No respondents felt that these promises of toxicogenomics were near. Two individuals mentioned related concepts that were far in the future: the ability to generate complete proteomic profiles and the ability to use the entire genome in gene expression microarrays.

Finally, metabonomics and metabolomics were singled out as approaches not yet ready for full implementation. The respondents noted that the technologies to make comprehensive analyses of metabolites were in relatively early stages of development, and that therefore the ability to standardize and analyze the data from these types of experiments was just now developing. The inability to perform comprehensive metabolite assays was related to the difficulty of developing complex biologic systems models.

Several concepts or applications were mentioned by only one or two respondents. One respondent opined that the ability to intervene clinically to address early signs of toxicologic insult was far off. Other one-time mentions included interspecies extrapolation and understanding the mechanisms of neurotoxicity.

### Question 5—What are the main barriers to implementation?

Respondents offered numerous answers to this question. Their responses could be separated into those that were primarily technical, relating either to the development of the laboratory technology itself or to the analysis and interpretation of data, and those that were primarily sociopolitical, including barriers within the community of scientists and regulators developing toxicogenomics as well as barriers within society at large.

One frequently mentioned technical barrier was the high cost of generating the data necessary to understand, standardize, and validate toxicogenomics results and methods. There was some disagreement as to whether the government was investing sufficient financial resources in toxicogenomics research to meet this need, but there was considerable agreement that a massive amount of data needs to be generated in the early stages of implementation to provide the understanding and context necessary for reliably interpreting individual experiments.

Related to this, many respondents noted that the amount of data generated by the toxicogenomics experiments was itself a barrier to the development and standardization of methods. Many mentioned that the methods, software, and hardware necessary to handle the massive amounts of data were being developed but were relatively new to most molecular biologists. In addition to the sheer quantity of data generated by toxicogenomics assays, many respondents also expressed concerns that the quality of data was a barrier. Respondents expressed concern about appropriate study design, the reproducibility of results across different laboratories and the different assays and reagents used.

A critical, frequently mentioned technical barrier was the ability to separate important changes from background “noise.” Different aspects of this issue include the need to distinguish between important signals of a much smaller magnitude than some benign changes in gene and protein expression that might occur simultaneously. In addition, changes are being monitored in tens of thousands of genes and hundreds of proteins at once, increasing the statistical probability of many false positives and false negatives. The difficulty in identifying true responses has underlined the particular importance of negative controls in toxicogenomics research. These challenges make the central task of defining significant changes in gene and protein expression extremely complicated.

In addition to these technical barriers, the respondents mentioned numerous sociopolitical barriers. Some of these were intertwined with the technical details of toxicogenomics and had to do with scientific culture and the nature of the research and regulatory institutions involved. For example, one frequently mentioned barrier is the difficulty in achieving the effective multidisciplinary collaboration needed to develop toxicogenomics effectively and appropriately. This problem is not unique to toxicogenomics and has always been a problem for environmental health and safety, but certain aspects of toxicogenomics were highlighted as posing special challenges because of the need for multidisciplinary work. For example, several respondents noted that the quantity and complexity of data analysis requires more effective collaboration between molecular biologists and computational biologists. Some respondents addressed this issue by requiring training molecular biologists to learn the programming that is necessary to analyze their own data; others emphasized multi-disciplinary input into study design to avoid asking the wrong questions and failing to generate the types of data needed to answer specific questions.

Difficulty of persuading the scientific community to change its methods for approaching problems was a second sociopolitical barrier. Several respondents pointed out that toxicologists tend to be conservative, to adhere to “tried and true” laboratory methods (i.e., traditional animal tests), and to be very slow in adopting new methods. A few respondents noted that this is especially true for regulatory toxicologists employed in government. A philosophical challenge constituted a more complex barrier. Several respondents spoke of moving from a paradigm of reductionism, in which scientists focus intently on one gene or one mechanism to solve problems, to a broader paradigm of complex biologic systems. Several stated that in making this paradigm shift, scientists would need to abandon the comfort of feeling that they understand in detail what changes are occurring in a gene or other component of a cell and instead rely on computerized analysis of changes in thousands of genes and hundreds of proteins. Both the resistance to change and the philosophical shift required were common themes in the interviews.

Turning from the scientific community to society at large, many respondents voiced concerns that societal forces would hinder the implementation and use of toxicogenomics advances. The most frequently mentioned was the concern that the development of societal mechanisms for addressing the ethical, legal, and social aspects of toxicogenomics (and toxicogenetics) was lagging behind the technologic advances. This was mentioned in two very different contexts. Many respondents expressed concern that privacy concerns and mistrust among the general public would impede the development of methods to determine and understand susceptibility. Others pointed to the lack of legal and regulatory mechanisms that allow the increased use of toxicogenomics data in regulatory matters. Several maintained that without clarity and some form of protection from premature regulatory use, industries would be reluctant to start generating and providing toxicogenomics data in new chemical or drug applications. They pointed out that the U.S. Food and Drug Administration was well ahead of the U.S. Environmental Protection Agency (EPA) and had developed a safe-harbor system that was indeed facilitating the development of genomics and proteomics data in new drug applications. Several respondents mentioned the related concept of incentives, noting that the chemical industry had little incentive to spend money to develop toxicogenomics data, that the pharmaceutical industry had little incentive to share databases, and that the health care industry in general had little incentive to make a large investment in research that would lead to prevention rather than therapeutics.

Many respondents’ comments were more appropriately categorized as ways of reducing barriers rather than identifying the barriers themselves. One respondent observed that the private sector, with its greater flexibility and focus, might be better suited to foster multi-disciplinarity. Several comments addressed steps the government needed to take, specifically the U.S. EPA. Suggestions included the need to invest more in data generation now that methods for gene arrays have been established and the need to train and develop U.S. EPA staff to be able to understand and use these data better. One respondent focused on the need to fund work that would better integrate data generation and the bioinformatics work needed to interpret the data. Last, several respondents emphasized the need for better educating toxicologists and scientists (and recruiting scientists already familiar with toxicogenomics to do that educating) as well as educating the general public before toxicogenomics is applied more widely.

### Question 6—How will the development of toxicogenomics affect the use of animals for testing?

There was a moderate amount of disagreement about how the development of toxicogenomics would affect the use of animals in testing in both the short and the long term. Most respondents felt that the use of animals would not decrease over the short term; some believed that their use would not be much affected; and others predicted an increase, perhaps sizable. Over the long term, more respondents felt that overall use of animals would decrease, although some predicted that the use of animals in testing would still be necessary, even as toxicogenomics methods supplied ever-greater amounts of information. In general, most respondents agreed that the promise of toxicogenomics to reduce the use of animals in testing would not be realized in the near term, and none felt that *in vitro* toxicogenomics methods would ever completely replace animals in toxicologic testing.

### Question 7—What are appropriate roles for the environmental public-interest community?

The most common response to this question was some form of public education. Many respondents felt that a critical role of the environmental public-interest community was to provide reliable information to the public about the benefits and limitations of toxicogenomics. Many respondents, particularly those from the academic and private sectors, emphasized the need to “paint an even picture” and to avoid overselling the potential of toxicogenomics. Respondents from the nonprofit sector emphasized the need to promote development of toxicogenomics applications as a means of improving our ability to detect toxicity and regulate toxic chemicals.

The second most common response involved science advocacy, either advocating for adequate funding or ensuring that the interpretation of results would be both scientifically rigorous and in the public interest. Some respondents mentioned the need to persuade government agencies to conduct the extensive basic science research required at this stage, whereas others stressed the need to engage policymakers in setting research agendas and ensuring that basic science research served policy needs. Many respondents emphasized the need for the public-interest community to be represented by scientists familiar with the technology and science issues so that the public-interest community could be part of the discussions between government agencies and industry and could play a watchdog role in those discussions. This was true for both the science-monitoring and the regulatory-monitoring themes. Several respondents underscored this by pointing to the need to provide a counterpoint to industry’s influence on the process of determining how toxicogenomics information would be incorporated into risk assessment and regulatory processes.

Finally, many respondents mentioned the need for a brokering function among industry interests, academics, and government staff, particularly with regard to the ethical, legal, and social implications of toxicogenomics. Several respondents cited the need to convene a workshop on this topic, led by public-interest groups.

## Discussion

Interviewees expressed widespread agreement that the new toxicology promises a significant increase in the amount of information available on toxic effects of chemicals. Nearly all respondents felt this would ultimately make it easier to identify and predict which chemicals cause adverse effects at environmentally relevant doses. And nearly all welcomed this improved ability because they were frustrated by the limitations of current toxicologic test batteries. For some, the main frustration was the limited ability of current toxicologic test batteries to assess subtle forms of toxicity that may occur at low doses; for others, the main source of frustration was the sense that extrapolations across dose and species leads to overly cautious standard setting. It is critical that discussion of implementing the new toxicology not get bogged down in a false dichotomy of whether the new will replace the old. Instead, the focus should be on how scientists and regulators can obtain the most information from these techniques now and how best to incorporate results from the new techniques in a progressive fashion that builds the experience necessary to make full use of this additional knowledge in the future.

The interviews show that the promise of the new toxicology will be realized only if a set of obstacles can be overcome. Respondents differed as to which obstacles were the most important and how quickly they might be overcome. Many of the obstacles identified were technical in nature, whereas others were political in nature. Public-interest scientists have a role to play in overcoming both types of obstacles. Advocacy for increased research resources will help address technical obstacles. Sociopolitical obstacles must be addressed through public and policymaker education and engagement in committees and stakeholder processes.

The most commonly identified technical needs were refining computational methods to be able to analyze vast, complex data sets, generating sufficient data to be able to train predictive toxicology models, and developing higher throughput assays for proteins and metabolites. Validation of assays and development of standardized data reporting frameworks were mentioned by some as significant obstacles, but other experts felt that such significant progress had been made in those areas that they were no longer major problems, at least for genomic data.

A recurrent sociopolitical obstacle mentioned by experts from many sectors was the inherent inertia of current toxicologic practices. This related not only to the reluctance of toxicologists and regulators to educate themselves about new toxicologic methods but also the reluctance of scientists to rely more heavily on computational analyses of complex patterns of responses that cannot easily be understood in terms of known mechanisms and pathways. This conflict has been played out in the literature in the context of clinical diagnostics and will likely become more prominent in debates about types of toxicogenomics data that can be used in regulatory settings (Diamandis et al. 2003). Along with phenotypic anchoring to existing end points, it is essential that the use of toxicogenomics data in a screening or regulatory setting not depend exclusively on achieving complete understanding of the functional implications of individual gene, protein, or metabolite changes. Although scientific rigor is necessary for the new toxicology to move forward, the scientific and public-interest communities must ensure that calls for rigor are not part of a strategy of foot-dragging and stalling.

The issue of proprietary databases was controversial. Several government and academic respondents maintained that information produced by biotechnology companies is often their sole commodity and thus there are strong disincentives to full sharing of new and useful data. Most respondents from the private sector downplayed this limitation, saying that the private sector was contributing significantly to open international databases and that the amount of data withheld for proprietary reasons was small. Landmark initiatives such as the Chemical Effects in Biological Systems knowledge base (http://cebs.niehs.nih.gov/) sponsored by the National Institute of Environmental Health Sciences will be limited in their value if only a subset of developed data are submitted to it. Clearly, this is an issue that must be monitored by both the public and the public-interest sectors.

The public-interest community has a critical role in helping guide the application of this powerful new science. As with all new technologies, societal risks may accompany societal benefits. Toxicogenomics promises to increase knowledge of biologic mechanisms and reduce toxicologic uncertainty. However, public-interest groups must engage in the science-policy process to ensure that needless barriers are not erected, that shortcuts are not taken, and that public-health protection and an individual’s privacy are not compromised. To engage effectively, public-interest groups must increase their capability in this new, exciting, and complex toxicology.
